# *Slc2a6* regulates myoblast differentiation by targeting LDHB

**DOI:** 10.1186/s12964-022-00915-2

**Published:** 2022-07-18

**Authors:** Xuan Jiang, Ninghan Feng, Yizhou Zhou, Xianlong Ye, Rong Wang, Jingwei Zhang, Siyuan Cui, Siyu Ji, Yongquan Chen, Shenglong Zhu

**Affiliations:** 1 Wuxi School of Medicine, Jiangnan University, 1800 Lihu Road, Wuxi, 214122 Jiangsu China; 2 Wuxi Translational Medicine Research Center, School of Translational Medicine, Jiangnan University, Wuxi, 214122 China; 3 School of Food Science and Technology, Jiangnan University, Wuxi, China; 4 Department of Urology, The Wuxi No. 2 People’s Hospital, Wuxi, 214002 China; 5 Ganjiang Chinese Medicine Innovation Center, Nanchang, 330000 China

**Keywords:** T2DM, Myogenesis, Slc2a6, Lactate

## Abstract

**Background:**

Type 2 diabetes mellitus is a global health problem. It often leads to a decline in the differentiation capacity of myoblasts and progressive loss of muscle mass, which in turn results in deterioration of skeletal muscle function. However, effective therapies against skeletal muscle diseases are unavailable.

**Methods:**

Skeletal muscle mass and differentiation ability were determined in db/+ and db/db mice. Transcriptomics and metabolomics approaches were used to explore the genetic mechanism regulating myoblast differentiation in C2C12 myoblasts.

**Results:**

In this study, the relatively uncharacterized solute carrier family gene *Slc2a6* was found significantly up-regulated during myogenic differentiation and down-regulated during diabetes-induced muscle atrophy. Moreover, RNAi of *Slc2a6* impaired the differentiation and myotube formation of C2C12 myoblasts. Both metabolomics and RNA-seq analyses showed that the significantly differentially expressed genes (e.g., LDHB) and metabolites (e.g., Lactate) during the myogenic differentiation of C2C12 myoblasts post-*Slc2a6*-RNAi were enriched in the glycolysis pathway. Furthermore, we show that Slc2a6 regulates the myogenic differentiation of C2C12 myoblasts partly through the glycolysis pathway by targeting LDHB, which affects lactic acid accumulation.

**Conclusion:**

Our study broadens the understanding of myogenic differentiation and offers the *Slc2a6*-LDHB axis as a potential therapeutic target for the treatment of diabetes-associated muscle atrophy.

**Video abstract**

**Supplementary Information:**

The online version contains supplementary material available at 10.1186/s12964-022-00915-2.

## Background

Type 2 diabetes mellitus (T2DM) is a metabolic disorder characterized by chronic hyperinsulinemia, hyperglycemia, and insulin resistance and has become an epidemic health threat and economic burden [1]. A major metabolic defect associated with T2DM is the failure of the proper glucose utilization by the skeletal muscle, which is the primary target site of insulin-stimulated glucose uptake. The skeletal muscle is the largest organ in the human body and comprises approximately 40–50% of the total body mass [2]. In addition, it is one of the major tissues responsible for glucose homeostasis, as approximately 80% of the glucose utilization occurs in the skeletal muscle [3]. Moreover, an increasing number of studies have demonstrated that T2DM causes dramatic structural, metabolic, and functional changes in skeletal muscle fibers, such as muscle atrophy [4], fiber-type transition [5], decreased myogenic differentiation ability, impaired glucose uptake [6], and fatty acid oxidation [7]. Skeletal muscle dysfunction, together with other diabetic complications, seriously affects the quality of life and physical activity of patients and increases the risk of death [8, 9]. The maintenance of skeletal muscle mass and integrity is regulated by myogenesis, which involves satellite-cell activation, myoblast differentiation, and myotube formation [10]. Moreover, myogenesis is regulated by myogenic regulatory factors, including myogenin (MyoG), myostatin, myogenic factor 5, myogenic regulatory factor 4, and myogenic differentiation (MyoD). Of these, MyoD and MyoG are essential in skeletal muscle development [11, 12]. Therefore, strategies that can improve skeletal muscle function by regulating myogenesis may promote the glucose uptake and metabolism in skeletal muscle cells, which could be useful for treating patients with T2DM. However, the myogenic differentiation process involves numerous pathways, and many potential targets for modulating myogenic differentiation are yet to be discovered.

The solute carrier (SLC) superfamily comprises > 400 transport proteins mediating the influx and efflux of various substances, such as ions, nucleotides, and sugars, across biological membranes [13]. At least half of the SLC transporters in humans are associated with diseases, including T2DM [14]. Dysfunction of skeletal muscle transport of small molecules such as glucose is the main reason for the occurrence and development of diabetes. Moreover, SLC transporters are overexpressed in metabolically active organs, such as skeletal muscle, and are considered promising novel drug targets in the treatment of T2DM and associated metabolic disorders [15, 16]. However, only a few SLC proteins are validated drug targets.

In this study, we conducted transcriptomics analyses in a classical myogenic differentiation model. We found that *Slc2a6* (also known as glut6) participates in the regulation of skeletal muscle myogenesis. Additionally, we show that *Slc2a6* regulates muscle development by targeting the glycolysis-related gene LDHB. Thus, this newly discovered *Slc2a6*-LDHB pathway may serve as an attractive therapeutic target against the sarcopenia caused by T2DM.

## Materials and methods

### Animal studies

Male C57BL KS/J-db/db mice (6–8 weeks old, n = 6) and their age-matched normoglycemic db/+ littermates (n = 6) were purchased from the Nanjing University Animal Model Research Center (Nanjing, China). All the mice were individually housed in a 12 h light–12 h dark cycle in a temperature-controlled room. Bodyweight was measured weekly, and blood glucose levels were determined using a glucometer (Roche). The mice were sacrificed at 12 weeks of age, and blood was collected from the orbital venous plexus. The gastrocnemius muscles were harvested, weighed, immediately flash-frozen using liquid nitrogen, and then stored at − 80 °C for further analysis. This study was conducted under the approval of the Ethical Committee of Jiangnan University, and all the procedures involving mice were carried out in accordance with the guidelines of the Institutional Animal Care and Use Committee of Jiangnan University (Approval Number: JN.No 2020915c0400910[192]).

### Cell culture and treatments

The C2C12 mouse myoblast cell line (ATCC®, CRL-1772™) was cultured in a growth medium (GM) of Dulbecco’s modified Eagle’s medium (DMEM, C11965500BT, Gibco, USA) with 10% fetal bovine serum (10099-141, Gibco, USA) and 1% penicillin–streptomycin (C0222, Beyotime, China) at 37 °C with 5% CO_2_. When cell confluence reached 80–90%, the GM was replaced with a differentiation medium (DM) of DMEM containing 2% horse serum (SH30074.02, Hyclone, USA) and 1% penicillin–streptomycin. Cells were fed with fresh DM every other day, and cultures were maintained for 5 d. In some experiments, cells were continuously incubated for 5 d in the DM with sodium lactate (0, 10, or 20 mM) purchased from Sigma-Aldrich. The concentration of lactate was set at 0–20 mM based on previous studies using skeletal muscle cells, and this range corresponds to the physiological range of blood lactate level in humans after an exercise [17]. Pyruvate or lactate was assessed using a lactic-acid assay kit or pyruvate assay kit (A019-2-1, A081-1-1, Nanjing Jiancheng Bio, China).

### Transfection of cells with small interfering RNAs (siRNAs)

The siRNAs against mouse Slc2a6, mouse LDHB and control NC were synthesized by GenePharma (Shanghai, China). C2C12 myotubes were transfected with 50 nM siRNA by using the JetPrime® transfection reagent (114-15, Polyplus Transfection, New York, NY, USA) according to the manufacturer’s protocol. After 6 h of transfection, the cell medium was replaced with the DM. The cells were cultured for 5 d and then collected for western blot or reverse transcription-quantitative polymerase chain reaction (qPCR) analysis. The siRNA sequences were as follows: Mouse Slc2a6-1: 5′-AGCCAUUGGCUAUGCAAUCAUTT-3′; Mouse Slc2a6-2: 5′-GUGUACGUGUCUGAGAUUGCATT-3′; Mouse LDHB-1: 5′-CGUCAUCAAUCAGAAGCUGAATT-3′; Mouse LDHB-2: 5′-CCCAGUGGAUAUUCUGACUUATT-3′.

### RNA extraction and qPCR analysis

Total RNA was extracted from C2C12 myoblasts and gastrocnemius samples by using TRIzol reagent (15596026, Invitrogen, USA). A total of 1 μg RNA was reverse transcribed into cDNA by using the PrimeScript™ RT Kit (Takara, Kyoto, Japan), and the SYBR Green Real-time PCR Master Mix reagent (Takara, Kyoto, Japan) was used for qPCR. The PCR reactions were carried out on a CFX96™ Optical Reaction Module (Bio-Rad, Hercules, CA, United States). The relative levels of target mRNAs were normalized to that of β-actin through the 2^−ΔΔCT^ method. The primers used are listed in Table 1.

**Table 1:** List of genes, primer sequences in this study

Gene name	Forward (5′-3′)	Reverse (5′-3′)
Myf5	AAGGCTCCTGTATCCCCTCAC	TGACCTTCTTCAGGCGTCTAC
MyoD	CCACTCCGGGACATAGACTTG	AAAAGCGCAGGTCTGGTGAG
MyoG	GAGACATCCCCCTATTTCTACCA	GCTCAGTCCGCTCATAGCC
MyHC	ACTGTCAACACTAAGAGGGTCA	TTGGATGATTTGATCTTCCAGGG
Slc2a6	AACCGAGGGACTCGACTATGA	CAAGGCATACCCAAAGCTGAA
PDHB	CGGTGCAGTTGACAGTTCGT	TCTTCCCCAAGCAGAAAAACTTT
LDHA	TGTCTCCAGCAAAGACTACTGT	GACTGTACTTGACAATGTTGGGA
LDHB	TGCGTCCGTTGCAGATGAT	TTTCGGAGTCTGGAGGAACAA
MCT1	TGTTAGTCGGAGCCTTCATTTC	CACTGGTCGTTGCACTGAATA
MCT4	TCACGGGTTTCTCCTACGC	GCCAAAGCGGTTCACACAC
β-actin	GATCTGGCACCACACCTTCT	GGGGTGTTGAAGGTCTCAAA

### Western blot analysis

Cells were lysed using radioimmunoprecipitation assay (RIPA) buffer (20 mM Tris- HCl, 1%SDS, 150 mM NaCl, 1% sodium deoxycholate, and 1% Triton X-100) containing 1 mM PMSF and 0.02% protease phosphatase inhibitors. The protein concentration of each lysate was evaluated using a bicinchoninic acid (BCA) protein assay kit (PC0020, Solarbio, Beijing, China). The proteins were resolved using sodium dodecyl sulfate–polyacrylamide gel electrophoresis (SDS-PAGE) and then transferred onto a polyvinylidene fluoride (PVDF) membrane. Subsequently, the membrane was blocked for 2 h at room temperature with tris-buffered saline containing Tween 20 (TBST) and 5% skim milk, and then incubated overnight at 4 °C with a primary antibody targeting Slc2a6 (ab119272, 1:2000, Abcam), MyoG (ab1835, 1:2000, Abcam), MyoD (18943-1-AP, 1:1,000, Proteintech), MyHC (MF-20, 1:1,000, DSHB), or β-actin (AC021, 1:5000, ABclonal Biotech). Lastly, the membrane was incubated with a goat anti-mouse (AS003, 1:5000, ABclonal Biotech) or anti-rabbit secondary (AS014, 1:5000, ABclonal Biotech) antibody for 2 h. All the blots were visualized using an ECL reagent (WBKLS0500, Millipore, USA), and the image was captured using a chemiluminescent imaging system (TanonSciences and Technology Co., Ltd., Shanghai, China). Band intensity was quantified using the Image J software (National Institutes of Health, Bethesda, USA). The expression level of each target protein was normalized to that of the internal control β-actin.

### Immunofluorescence assay

C2C12 myoblast differentiation was evaluated by immunofluorescence staining of skeletal muscle myosin heavy chain. After siRNA transfection or lactate treatment, the myoblasts were induced to undergo differentiation for 5 d in 12-well plates. Afterward, the cells were washed three times with PBS, fixed in 4% paraformaldehyde for 15 min, permeabilized with 0.5% Triton X-100 for 15 min, and then washed again with PBS. Subsequently, they were incubated with goat serum (Solarbio, Beijing, China) at room temperature for 1 h and then with anti-MyHC (MF-20, 1:50, DSHB) at 4 °C overnight. Afterward, they were incubated with the FITC-conjugated anti-goat IgG antibody (1:100, ABclonal Biotech) at room temperature for 2 h. DAPI (C1002, Beyotime, China) was used to detect cell nuclei. A Nikon Eclipse Ti-U fluorescence microscope was used to capture fluorescence images. At least five views were captured per sample. Myotube fusion index was calculated as the percentage of nuclei inside the MyHC-positive myotubes relative to the total number of nuclei, by using the Image J software (National Institutes of Health, Bethesda, USA).

### RNA-seq and bioinformatics analyses

RNA-seq was performed as previous described [18]. Briefly, to assess gene expression levels, total RNA was extracted from purified differentiating myoblasts by using TRIzol reagent (15596026, Invitrogen, USA). Libraries were prepared using the VAHTS total RNA-seq library prep Kit for Illumina (NR603, Vazyme, China). RNA-seq was performed by GENEWIZ (Suzhou, China) on an Illumina Hiseq 4000 platform (150 bp pair-end reads) according to the manufacturer’s protocol. The RNA-Seq raw data were processed through the standard RNA-Seq analysis pipeline. After genome mapping to GRCm38.p4 (mm10) by applying STAR (version: 2.5.3a), we normalized the clean reads to FPKM (fragments per kilobase of exon model per million mapped reads) by using the DESeq2 package in R language. Differentially expressed genes (DEGs) were identified based on the criteria of |log_2_ (fold change)|≥ 1 and *p*-value ≤ 0.05 and then subjected to comparative functional enrichment Kyoto Encyclopedia of Genes and Genomes (KEGG, http://www.kegg.jp) pathway analysis in Metascape (http://www.metascape.org/). A *p*-value < 0.05 denotes a significant KEGG pathway. For visualization, heatmap clustergram, Venn, and volcano plots were drawn using R (http://www.r-project.org/).

### Metabolite extraction and untargeted metabolomics analysis

C2C12 cells were induced to differentiate in 6-well plates for 5 d as described above. After washing the cells with 1 mL PBS, 400 μL cold methanol (− 20 °C) and 400 μL cold water was added to each well. They were then scraped using a cell scraper while maintaining the plates on ice. The resulting cell suspension was mixed with 200 μL of cold chloroform (− 20 °C), and the mixture was agitated for 20 min on a thermomixer set to 1,000 rpm and 4 °C. Subsequently, the samples were centrifuged at 13,000×*g* for 15 min at 4 °C, and then 800 μL of the upper layer (containing polar metabolites) was dried under vacuum at 4 °C. The dried extracts were derivatized by incubating them in 50 μL of 40 mg/mL methoxylamine in pyridine for 1 h on a thermomixer set to 37 °C. These samples were further derivatized with 50 μL of MSTFA + 1% TMCS at 70 °C for 1.5 h.

The derivatized extracts were analyzed via gas chromatography-mass spectrometry (GC–MS) by using TRACE™ 1310/TSQ8000Evo (Thermo Fisher Scientific™, Waltham, MA, USA). Briefly, the samples (1 μL per sample) were separated on an HP-5MS column (30 m × 0.25 mm × 0.25 μm, Agilent J&W Scientific, Folsom, CA, USA) at a flow rate of 1 mL/min with helium used as a carrier. The oven temperature was set to 60 °C for 2 min and then maintained at 310 °C for 6 min after increasing the temperature at a rate of 8 °C/min. The temperatures of the transfer line and ion source were maintained at 270 °C and 230 °C, respectively. Electrospray ionization in full-scan mode was conducted for MS detection, and the range of mass/charge (m/z) values was 50–600.

Peak identification of the GC–MS data was carried out using the Xcalibur 4.0® software (ThermoFisher Scientific) and the mass spectra with the NIST 11 library. The peak detection, deconvolution, and metabolite identification were performed using the TraceFinder® 4.1 software (ThermoFisher Scientific). SIMCA 14.0 (Umetrics, Sweden) was used for statistical analysis. Orthogonal projections to latent structures discriminant analysis (OPLS-DA) were used to distinguish the si-NC and si-Slc2a6 groups. A variable importance in projection (VIP) value > 1.0 and *p*-value < 0.05 were considered to indicate statistical significance. MetaboAnalyst 5.0 (http://www.metaboanalyst.ca) and the KEGG were used for pathway analysis.

### Statistical analysis

All the data were obtained from at least three independent experiments per treatment. The differences between groups were analyzed using Student’s *t*-test or one-way ANOVA and the GraphPad Prism 8.0 or SPSS software. Pearson’s correlation analysis was performed to evaluate the associations between sets of data. Data are presented as mean ± SEM. Statistical significance was achieved when the value of *p* < 0.05 (*p < 0.05, **p < 0.01, and ***p < 0.001).

## Results

### Skeletal muscle mass and differentiation ability are decreased in diabetic mice

**Fig. 1:**
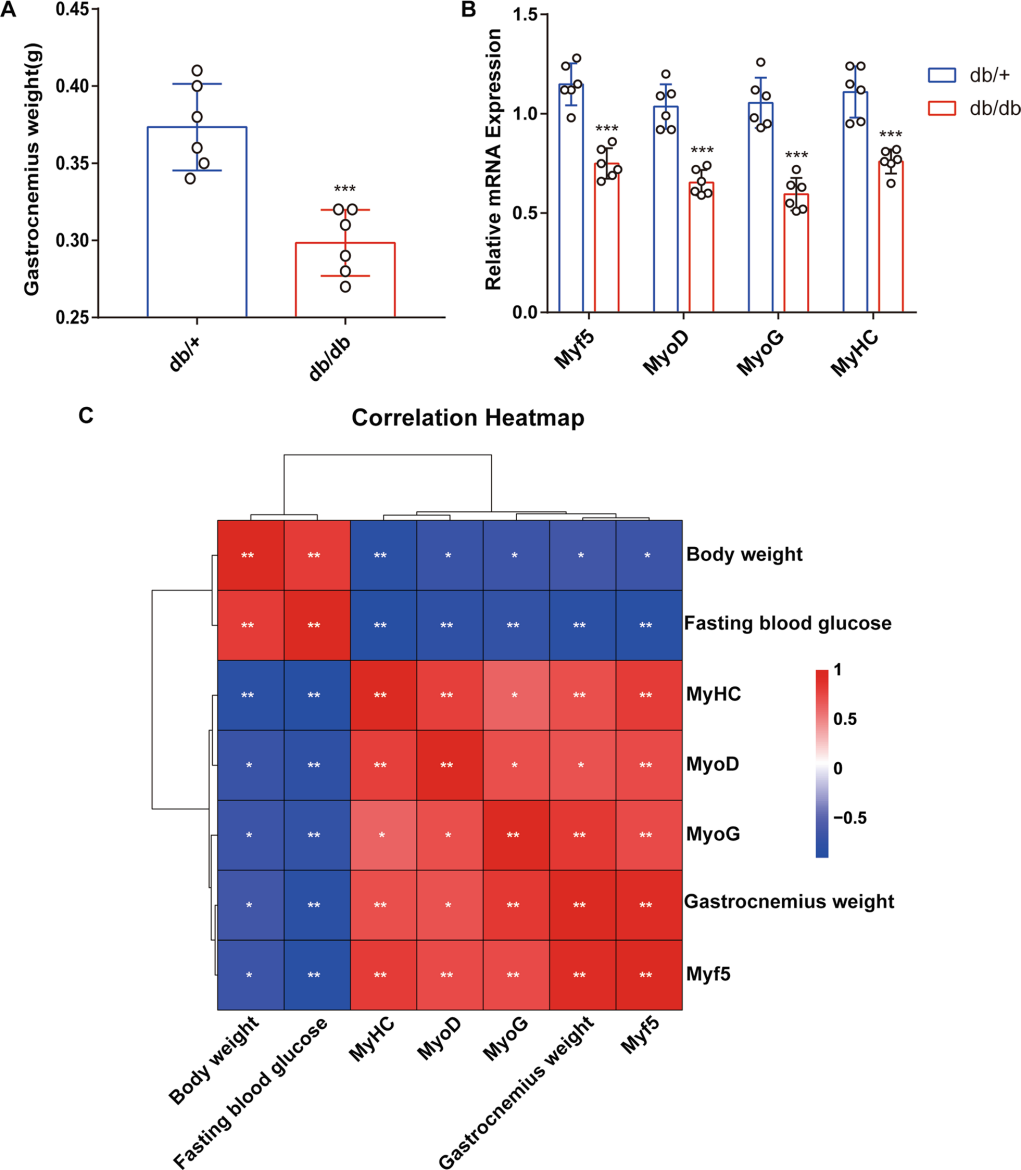
Correlation between diabetes and myogenic differentiation. **a** Gastrocnemius muscle weight (GW). **b** The mRNA levels of myogenesis-related genes in db/+ and db/db mice. **c** Correlation heatmap (Spearman correlation) of the body weight, fasting blood glucose level, gastrocnemius muscle weight, and mRNA levels of myogenesis-related genes. Red and blue colors indicate positive and negative correlations, respectively. The data are expressed as mean ± SEM, n = 6 per group. *p < 0.05, **p < 0.01, ***p < 0.001

To examine the effect of diabetes on skeletal muscle differentiation, we used db/db obese diabetic mice. Previous studies have shown that these mice develop severe obesity and diabetes and impaired muscle regeneration [19]. As presented in Additional file 1: Fig. S1a, b, body weight and fasting blood glucose level were significantly higher in db/db mice than in the non-diabetic db/+ mice. However, the db/db mice had a lower gastrocnemius muscle mass (Fig. 1a) than the db/+ mice. These observations suggest that diabetes can lead to muscle atrophy. Furthermore, compared with the db/+ mice, the gastrocnemius muscle from the diabetic mice showed a significant decrease in the mRNA levels of myogenesis-related genes, including Myf5, MyoD, MyoG, and MyHC (Fig. 1b). Based on Spearman’s correlation analysis, we assessed the correlation among the gastrocnemius muscle mass, expression levels of myogenesis-related genes, and body characteristics (body weight and fasting blood glucose level) in the diabetic mice (Fig. 1c). As expected, the gastrocnemius muscle mass and the expression levels of the myogenesis-related genes displayed a significant positive correlation. However, there was a significant negative correlation between the body characteristics and gastrocnemius muscle mass. Taken together, these results suggest that muscle mass and myogenesis capacity are decreased in diabetes.

### Slc2a6 is upregulated during C2C12 myoblast differentiation and downregulated in the skeletal muscle of diabetic mice

**Fig. 2:**
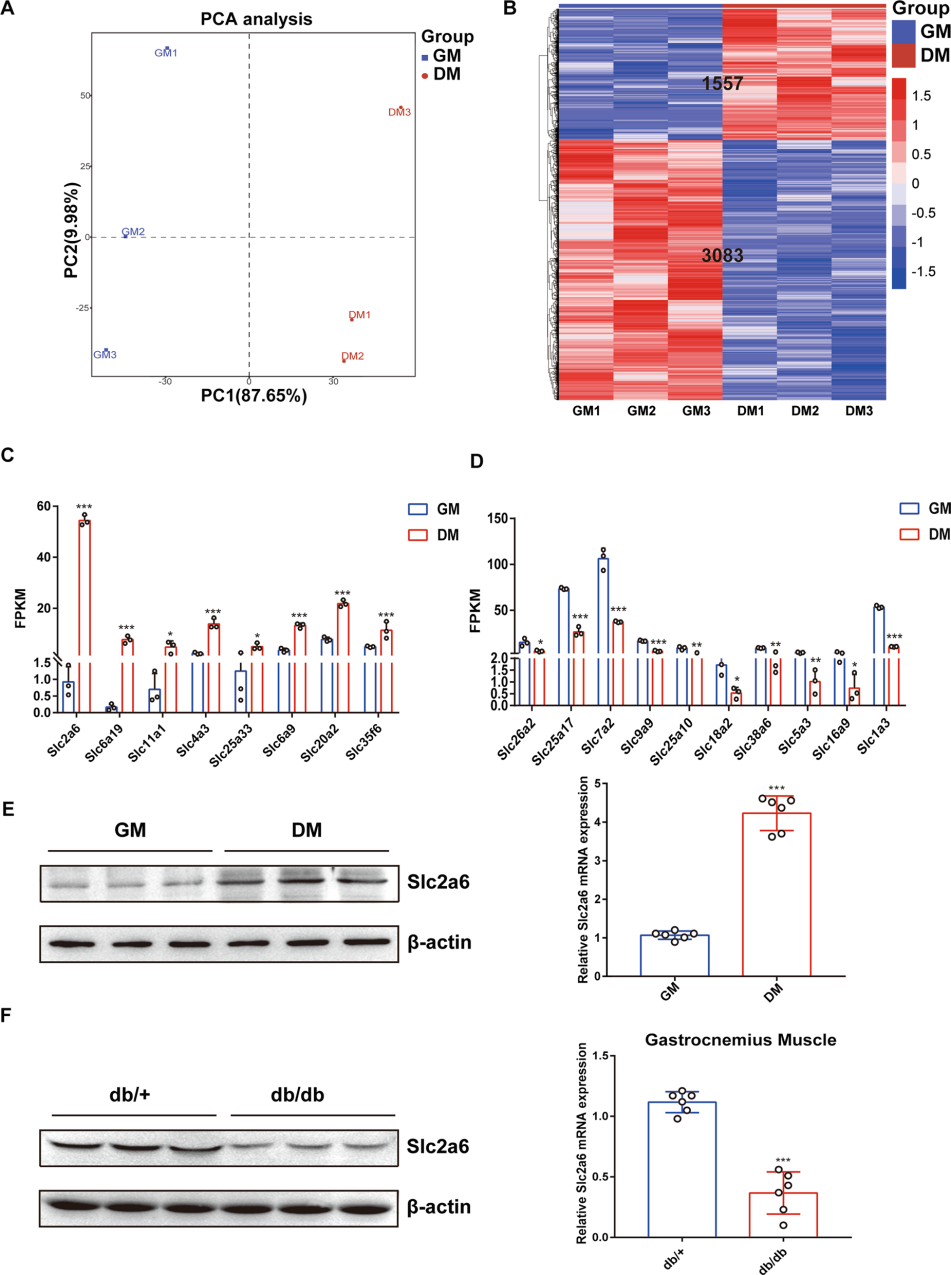
*Slc2a6* is upregulated during myogenesis. **a** Principal component analysis on the RNA-Seq data from cells in the GM and DM (n = 3 per group). **b** Heatmap and hierarchical clustering analysis of differentially expressed genes between the cells in the GM and those in the DM (n = 3 per group). FPKM bar plots of representative upregulated, **c** downregulated, **d** solute carrier family genes. **e** The protein level and mRNA expression level of *Slc2a6* in undifferentiated or differentiated C2C12 cells. **f** The protein level and mRNA expression level of *Slc2a6* in gastrocnemius muscle from db/+ or db/db mice. All the data are expressed as mean ± SEM, *p < 0.05, **p < 0.01, ***p < 0.001

To uncover potential genes that are involved in the regulation of myoblast differentiation, transcriptomics was performed comparing differentiated to undifferentiated C2C12. C2C12 is a myoblast cell line and is often utilized to study cellular differentiation and cell fusion to form myotubes [20, 21]. The DM induced the differentiation of C2C12 myoblasts into myotubes, with elongated and wide cylindrical shapes and multiple nuclei (Additional file 2: Fig. S2a). The MyoD, MyoG, and MyHC protein levels in the cells were markedly increased during the myogenic differentiation (Additional file 2: Fig. S2b). Principal component analysis (PCA) of the RNA-seq data was performed to understand the similarities and differences between the differentiated and undifferentiated cells. According to the score plots (Fig. 2a), the two major principal components explained 87.65% and 9.98% of the total variance, respectively. This result indicated that there were obvious differences in overall transcriptomic profiles between the two groups. Heatmap hierarchical clustering (Fig. 2b) showed that 1557 genes were differentially upregulated (Fold change > 1, *p* < 0.05) between the two groups, whereas 3083 genes were downregulated (Fold change > 1, *p* < 0.05). Furthermore, many myogenic genes were identified to be significantly up-regulated (Additional file 2: Fig. S2e). Meanwhile, among the differentially expressed genes, we found that many solute carrier family genes were significantly upregulated (Fig. 2c) or downregulated (Fig. 2d) upon myogenic differentiation**,** and the FPKM value of *Slc2a6* showed the most significant change (Fold change > 58, *p* < 0.001). During myogenic differentiation, up-regulated genes often play a role in promoting differentiation. Among these up-regulated differentially expressed solute carrier family genes, only Slc20a2 has previously been reported, and it was identified that Slc20a2 is essential for normal myofiber function and survival in hypophosphatemic myopathy [22].

The RNA-seq data was confirmed via RT-qPCR and Western blot, which showed that the *Slc2a6* protein and mRNA level was significantly upregulated after 5 days of differentiation (Fig. 2e), indicating that *Slc2a6* is involved in myogenesis. We also examined the *Slc2a6* protein and mRNA level in the diabetic db/db mice and found that *Slc2a6* was significantly downregulated in their gastrocnemius muscle compared with the level in the non-diabetic db/+ mice (Fig. 2f). Collectively, these results indicated that *Slc2a6* is significantly upregulated upon myogenesis but downregulated in T2DM.

### Silencing Slc2a6 prevents C2C12 myoblast differentiation

**Fig. 3:**
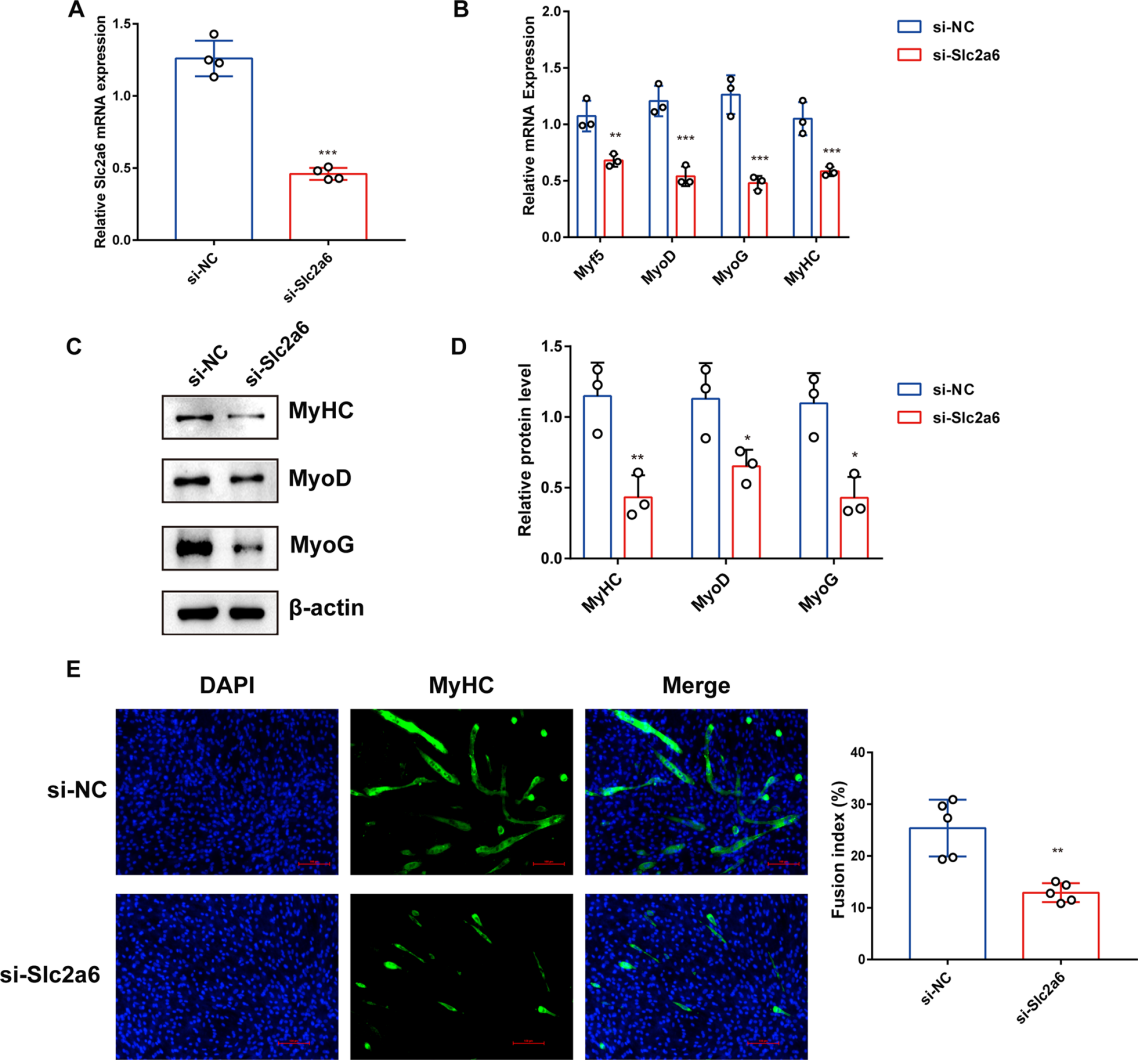
Knocking down *Slc2a6* inhibits C2C12 myoblast differentiation. **a** The *Slc2a6* mRNA levels in C2C12 cells transfected with a control siRNA (si-NC) or a siRNA against *Slc2a6* (si-*Slc2a6*) during myoblast differentiation. The mRNA levels were measured via reverse transcription–quantitative polymerase chain reaction after 5 d of transfection. **b** The mRNA levels of myogenic genes (Myf5, MyoD, MyoG, and MyHC) in si-NC– or si-Slc2a6-transfected cells. **c** Western blot analysis for MyHC, MyoD, MyoG, and β-actin. C2C12 cells were differentiated for 5 d. **d** Quantification of the western blot bands in **c** by using the Image J software with β-actin used for the normalization of the expression levels. The relative protein levels were obtained through western blot band gray scanning analysis. **e** Cells were stained with an anti-MyHC antibody and DAPI and observed via fluorescence microscopy (×200). Green, MyHC-positive cells; blue, DAPI-positive nuclei, Scale bar: 100 µm. Quantification of the fusion index (%) in **e**. The number of nuclei inside MyHC-positive myotubes was divided by the total number of nuclei in various fields. All the data are expressed as mean ± SEM from three separate experiments, *p < 0.05, **p < 0.01, ***p < 0.001 compared with the si-NC group via one-way ANOVA

To investigate the role of *Slc2a6* in the myogenic differentiation of C2C12 cells, *Slc2a6* was silenced during their differentiation. A siRNA targeting *Slc2a6* (si-*Slc2a6*) substantially downregulated the *Slc2a6* level in C2C12 cells (Fig. 3a). Moreover, the mRNA (Fig. 3b) and protein (Fig. 3c, d) levels of Myf5, MyHC, MyoD, and MyoG were significantly reduced upon *Slc2a6* knockdown compared with the levels in the si-NC group. We further examined the morphology of differentiated si-NC or si-*Slc2a6* treated C2C12 cells using an anti-MyHC antibody, *Slc2a6* knockdown significantly decreased the fusion index (number of nuclei inside MyHC-positive myotubes/total number of nuclei) compared with that of si-NC cells (Fig. 3e). Taken together, these data indicate that *Slc2a6* plays a positive regulatory role in the myogenic differentiation of C2C12 myoblasts.

### Transcriptomics analysis results implicate that Slc2a6 regulates glycolysis and gluconeogenesis

**Fig. 4:**
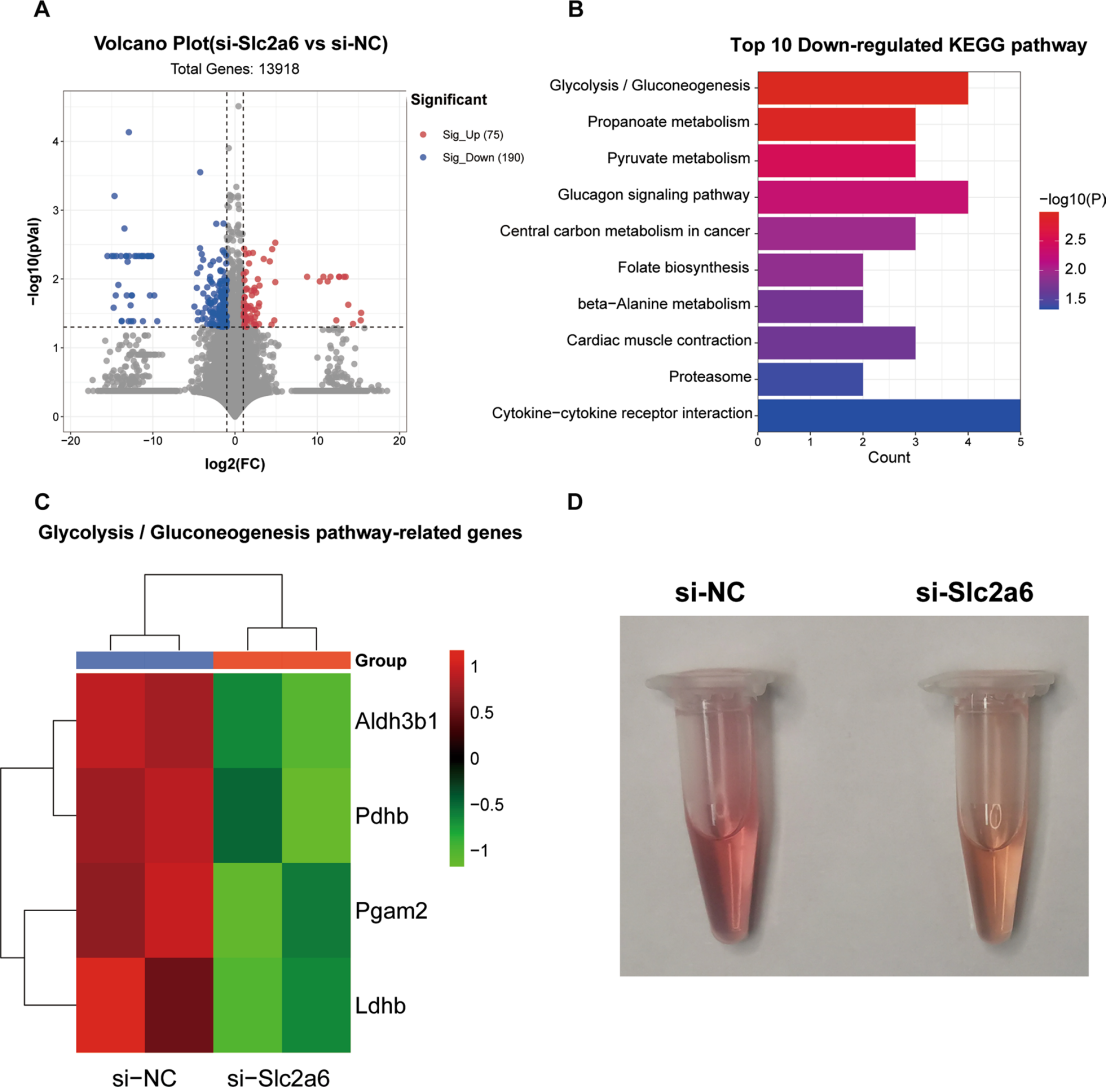
Transcriptomics analysis. **a** The volcano plot displays an overview of the differntially expressed genes (DEGs) between si-NC and si-*Slc2a6* group. The X-axis represents the log-transformed *p*-value, and the Y-axis indicates the multiple of the DEGs. The gray dots represent the DEGs that are not differentially expressed, the red dots represent the upregulated DEGs, and the blue dots represent the downregulated DEGs. |Log_2_ Fold Change| > 1 and *p* < 0.05 were set as the criteria. **b** The top 10 significantly downrelated KEGG pathways. The X-axis represents the number of DEGs enriched in a pathway. **c** Heatmap showing the differentially expressed genes involved in glycolysis/gluconeogenesis pathway. **d** The color of the culture media was photographed after knocking down Slc2a6. All the data are expressed as mean ± SEM from three separate experiments. *p < 0.05, **p < 0.01, ***p < 0.001, si-NC versus si-*Slc2a6* group

To explore the potential mechanism of *Slc2a6* in myoblast differentiation, the gene-expression profile of C2C12 cells with *Slc2a6* knockdown was compared with that of the si-NC cells. Based on the criteria of |log_2_ (fold change)|≥ 1 and *p*-value ≤ 0.05, we identified 265 DEGs between the two groups. Among these DEGs, 75 genes were upregulated and 190 genes were downregulated between the two groups (Fig. 4a). *Slc2a6* was markedly downregulated (by 88%, based on the level in the si-NC group). The top 10 significantly enriched pathways among the DEGs were determined via KEGG enrichment analysis (Fig. 4b). The glycolysis/gluconeogenesis pathway was the most enriched, followed by metabolic pathways, such as the propanoate metabolism, pyruvate metabolism, and cardiac muscle contraction pathways (Fig. 4b). Furthermore, we established Heatmap displayedthe aldehyde dehydrogenase 3 family member B1 (Aldh3b1), pyruvate dehydrogenase E1 subunit beta (Pdhb), phosphoglycerate mutase 2 (Pgam2), and lactate dehydrogenase B (Ldhb) genes, which participate in the glycolysis/gluconeogenesis pathway, were significantly downregulated after knockdown of Slc2a6 in C2C12 cells (Fig. 4c).

Interestingly, we observed that the color of the culture medium was changed from pink to yellow after transfecting C2C12 cells with si-*Slc2a6* compared with that of the si-NC cells (Fig. 4d), suggesting that *Slc2a6* knockdown resulted in the accumulation of acidic substances, which could affect the pH of the medium. Altogether, these results suggest that *Slc2a6* knockdown inhibits the myogenic differentiation of C2C12 cells by regulating the glycolysis and gluconeogenesis pathways.

### Metabolomic profiling reveals that lactic acid is upregulated upon Slc2a6 knockdown

**Fig. 5:**
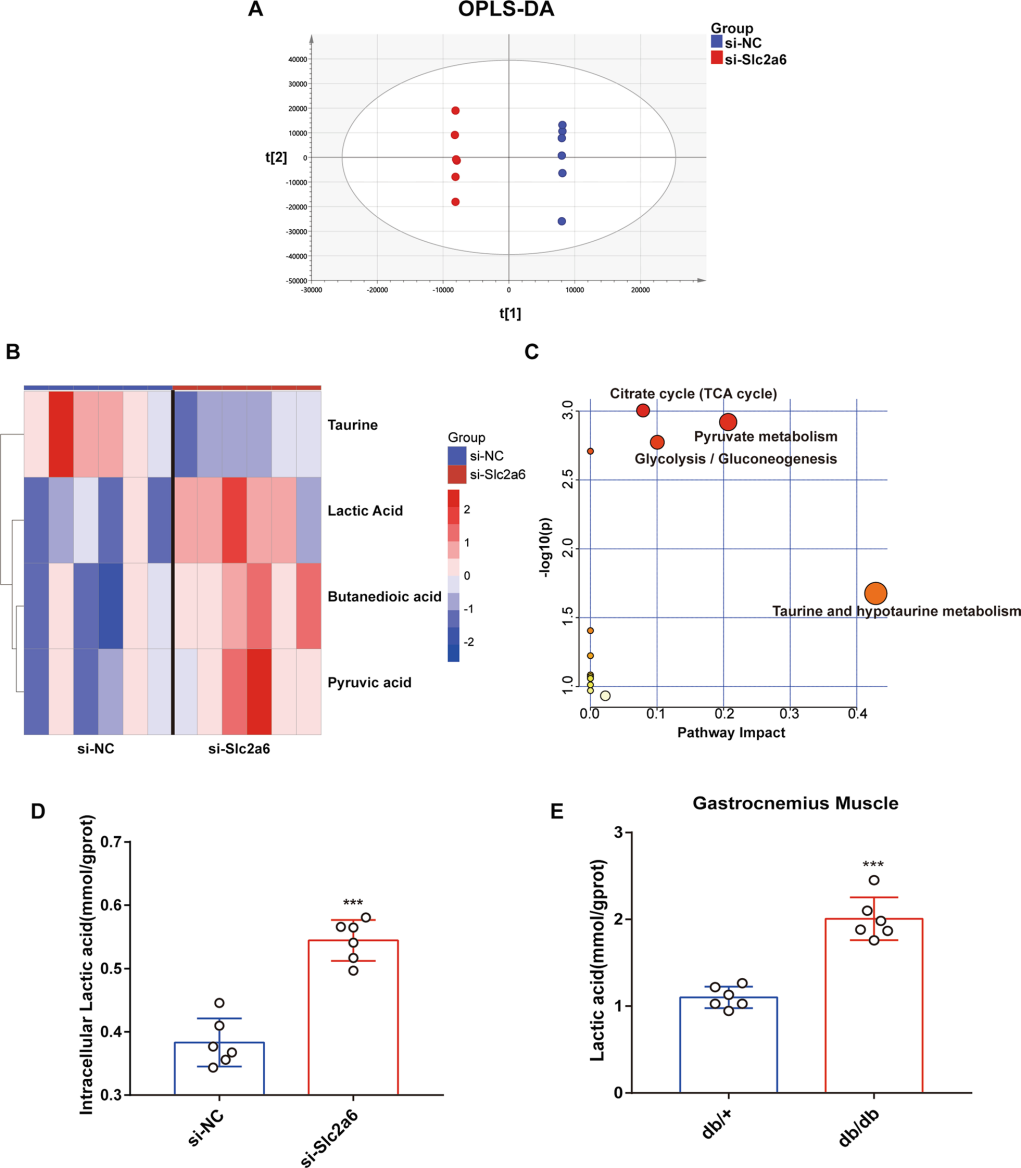
Untargeted metabolomics analysis. **a** Orthogonal partial least squares-discriminant analysis (OPLS-DA) of the differentially present metabolites, identified via GC–MS, in *Slc2a6*-knocked-down (si-*Slc2a6*) versus control (si-NC) C2C12 cells. **b** Heatmap showing these metabolites (VIP > 1, *p* < 0.05). The color of each section is proportional to the significance of the change (red, upregulated; blue, downregulated). Rows and columns correspond to the samples and metabolites, respectively. **c** The metabolic pathways in which the affected metabolites were involved, identified using MetaboAnalyst 5.0. **d** Intracellular and **e** gastrocnemius muscle lactic acid (mmol/gprot) levels in db/+ and db/db mice were measured using a lactic acid test kit. All the data are expressed as mean ± SEM from three separate experiments, n = 6 per group. *p < 0.05, **p < 0.01, ***p < 0.001, si-NC versus si-*Slc2a6* group

To explore the potential mechanism and factor through which *Slc2a6* regulates glycolysis and gluconeogenesis, we first performed an untargeted metabolomics analysis on the transfected cells via GC–MS to identify the active metabolites. Consequently, 151 metabolites were identified. As shown in Fig. 5a, a supervised OPLS-DA analysis was performed in the clustering of si-NC and si-*Slc2a6* groups, and the results suggested that metabolic profiles were different between the two groups. VIP and coefficients from OPLS-DA were used to identify significantly differential metabolites. As a result, lactic acid, butanedioic acid, and pyruvic acid were found significantly upregulated, and taurine tended to decrease in the *Slc2a6* knocked-down cells compared with the levels in the control group. Among these metabolites, lactic acid displayed the most significant fold change and *p*-value (Fold change = 1.68, *p*-value < 0.006) (Fig. 5b).

To identify the metabolic pathways associated with the compounds impacted by *Slc2a6* knockdown, the identified metabolites were matched with the known compounds in the KEGG and analyzed using MetaboAnalyst 5.0 (Fig. 5c). As mentioned above, pyruvate metabolism, glycolysis/gluconeogenesis, citrate cycle (TCA cycle), and taurine and hypotaurine metabolism were the main metabolic pathways notably affected after *Slc2a6* knockdown. According to the OPLS-DA results and the characteristics of the main metabolites, we hypothesized that lactic acid, as the product of glycolytic metabolism and the substrate of energy metabolism may be the key substances related to the medium color change after si-*Slc2a6*. Furthermore, we confirmed that the lactic acid levels in C2C12 cells, medium and in the skeletal muscle of db/db mice were significantly increased (Fig. 5d, e, Additional file 3: Fig. S3a). Meanwhile, we assessed the expression of the monocarboxylate transporter MCT1 and MCT4 by qPCR (Additional file 3: Fig. S3b), which are involved in lactate uptake and export, respectively. The result indicated that the mRNA expression level of MCT1 did not change, but the mRNA expression level of MCT4 was significantly up-regulated after *Slc2a6* knockdown. In summary, these data suggest that knocking down *Slc2a6* during C2C12 differentiation increased lactate production.

### Slc2a6 targets LDHB to regulate lactate metabolism

**Fig. 6:**
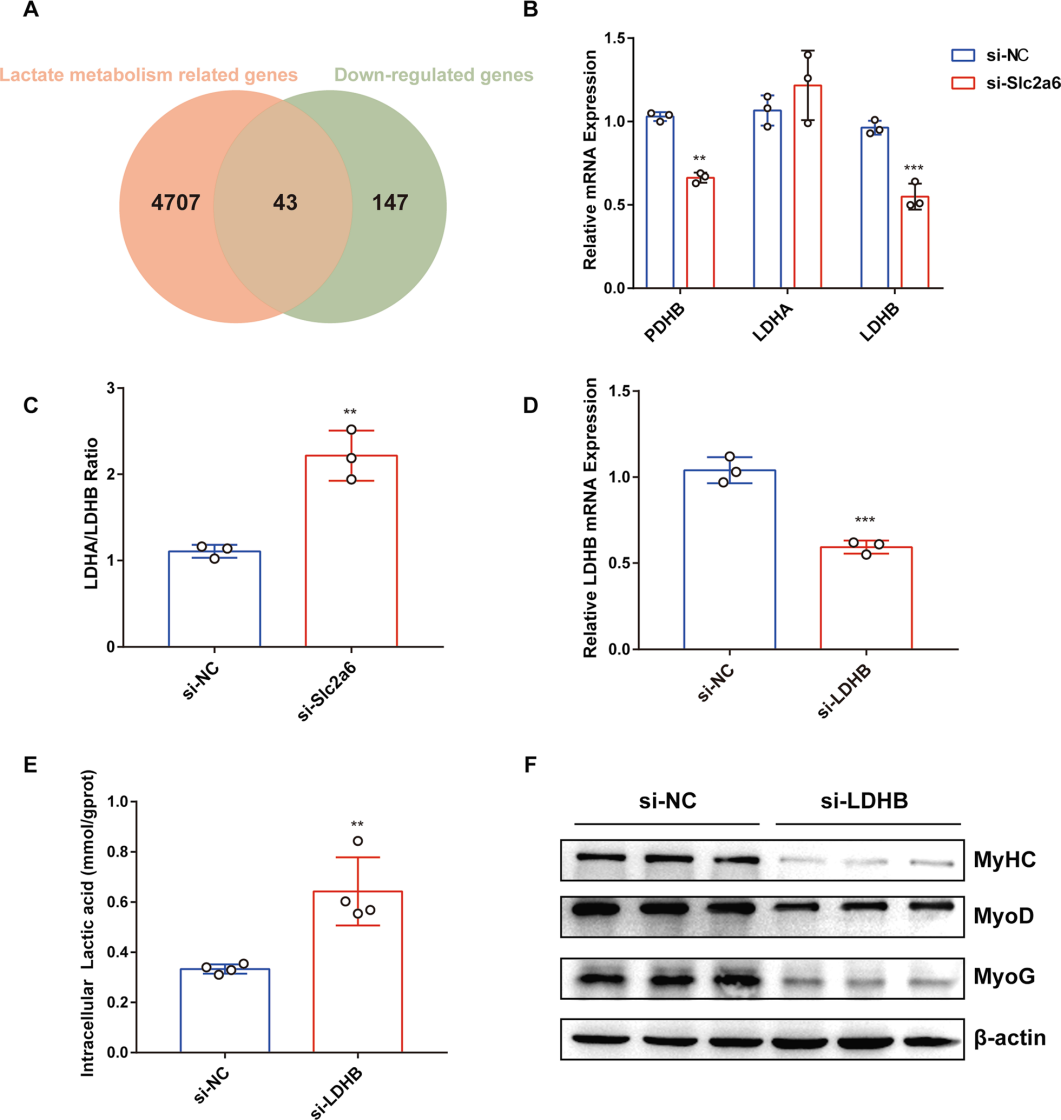
LDHB knockdown impairs C2C12 myoblast differentiation. **a** Venn plot of the genes related to lactic acid metabolism in Genecard datasets and the significantly downregulated genes based on the RNA-seq data. **b** The PDHB, LDHA, and LDHB mRNA levels in Slc2a6-knocked-down (si-*Slc2a6*) versus control (si-NC) C2C12 cells. **c** The ratio of LDHA mRNA level to that of LDHB. **d** Relative LDHB mRNA level after knocking down LDHB during C2C12 myoblast differentiation. **e** Intracellular lactic acid level (mmol/gprot) after LDHB knockdown. **f** Western blot analysis for the MyHC, MyoD, MyoG, and β-actin levels in the si-NC and si-LDHB groups. All the data are expressed as mean ± SEM from three separate experiments. *p < 0.05, **p < 0.01, ***p < 0.001

To determine which gene mediates the elevation in lactic acid level upon *Slc2a6* knockdown, we utilized a Venn diagram (Fig. 6a) to show the overlapping genes (43 genes) between Genecard datasets (4750 lactate-metabolism–related genes) and the RNA-seq data after *Slc2a6* knockdown (190 significantly downregulated genes). According to the relevance score calculated by the Genecard database, LDHB and PDHB were the top two genes (64.5 and 7.3, respectively). We then performed qPCR analysis and confirmed that the two genes were remarkably downregulated in the si-*Slc2a6* group compared with the levels in the si-NC group (Fig. 6b). PDHB breaks down pyruvate to acetyl-CoA and carbon dioxide and provides the primary link between glycolysis and the TCA cycle [23]. Our previous findings demonstrated that knocking down *Slc2a6* results in intracellular lactate accumulation. Lactate is produced by LDH, which forms a multiprotein complex consisting of LDHA and LDHB. LDHA catalyzes the conversion of pyruvate to lactate, and LDHB catalyzes the opposite reaction. Therefore, the ratio of LDHA/LDHB determines the dominant direction between pyruvate and lactate. We observed that knocking down Slc2a6 increased the LDHA/LDHB mRNA level ratio (Fig. 6c), and the LDHA mRNA level was not different between the si-NC and si-*Slc2a6* groups (Fig. 6b). These results indicated that LDHB may be the factor that regulated the lactic acid metabolism downstream of *Slc2a6*. Therefore, we then explored the impact of knocking down LDHB on C2C12 myoblast differentiation. Compared with the control level, the expression of LDHB was significantly reduced in the cells transfected with a siRNA targeting LDHB (Fig. 6d) in parallel to a significant increase in the lactic acid level (Fig. 6e). The content of intracellular pyruvate was significantly decreased after knockdown of LDHB (Additional file 3: Fig. S3c). In addition, western blotting results indicated that LDHB knockdown notably decreased the MyHC, MyoD, and MyoG protein levels (Fig. 6f). Thus, these results suggest that the lactic acid accumulation induced by Slc2a6 knockdown may be due to reduced LDHB expression, which reduces the transformation of lactic acid to pyruvate. In conclusion, *Slc2a6* may regulate glycolysis by targeting LDHB during C2C12 myoblast differentiation.

### Lactate inhibits myogenesis

**Fig. 7:**
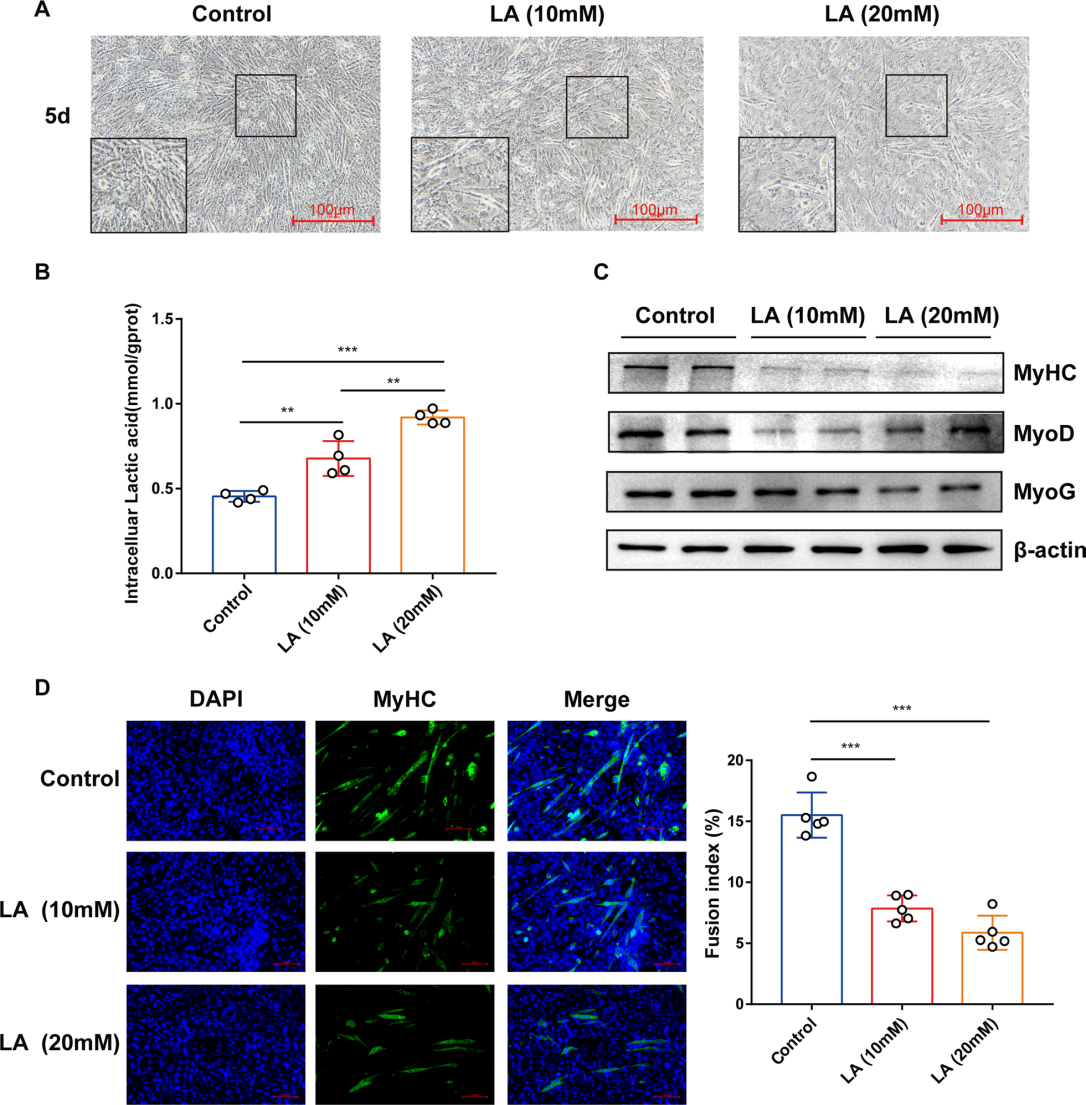
Lactate inhibits the myogenic differentiation of C2C12 cells. **a** Microscopic images of C2C12 myoblasts after the induction of their myogenic differentiation in the presence of 10 mM or 20 mM sodium lactate (LA) for 5 d. Scale bar, 100 µm. **b** Intracellular lactic acid level (mmol/gprot) after the LA treatment. **c** MyHC, MyoD, MyoG, and β-actin protein levels were determined using western blot analysis. **d** Representative images from MyHC (green) immunofluorescence analysis of C2C12 myotubes after the LA treatment (DAPI, blue). Scale bar, 100 µm. **e** The fusion index (%), defined as the percentage of nuclei located within MyHC-positive myotubes among the total nuclei, after the LA treatment was calculated as described in the methods section. All the data are expressed as mean ± SEM from three separate experiments. *p < 0.05, **p < 0.01, ***p < 0.001

The lactic acid level in the muscle has previously been shown to be higher in T2DM patients than in healthy individuals, and the accumulation of lactic acid in the muscle may be one of the reasons underlying the sarcopenia in these patients [24]. Therefore, we examined the impact of various lactate doses on myotube formation. The typical morphology of the myotubes is shown in Fig. 7a. We observed that the length, diameter, and number of myotubes formed from lactate-treated C2C12 cells were decreased compared with the levels in untreated cells. Moreover, the intracellular lactate level was increased after lactate treatment for 5 d compared with the level in the untreated group (Fig. 7b). In addition, we observed that the MyoD, MyoG, MyHC protein levels were notably decreased by lactate (Fig. 7c). Furthermore, immunocytochemical staining of the myotubes for MyHC and the fusion index (%) values showed that lactate overload (10 mM or 20 mM) significantly reduced the myotube formation and fusion, and 20 mM had the strongest effect (Fig. 7d)*.* Overall, these results demonstrated that lactate inhibits the differentiation of myoblasts.

## Discussion

Skeletal muscle myogenesis is a complex process, and the underlying mechanistic details are not fully understood yet. Here, we identified that a solute carrier family gene known as *Slc2a6* is a key regulator in myogenic differentiation.

The solute carrier family is an important protein class governing compound transport across biological membranes. *Slc2a6* encodes a recently identified solute carrier family 2-facilitated glucose transporter member 6 and contributes to glucose uptake in the multiple types of cells or organs [25]. In addition, *Slc2a6* is thought to be induced by inflammatory stimuli and modulate glycolytic activation in inflammatory macrophages [26]. In this study, we revealed that *Slc2a6* knockdown inhibited the myogenic differentiation of C2C12 cells, and glycolysis was the most significantly affected process in the knocked-down cells, which was closely connected to the biological function and metabolism of the skeletal muscle. Myogenic differentiation requires energy consumption to meet morphological changes. Knockdown of *Slc2a6* results in the inability of extracellular glucose to be transported into the cell. Meanwhile, intracellular glucose is consumed in large quantities, and the utilization of glucose produces a large amount of pyruvate. Pyruvate is primarily utilized through glycolysis and the tricarboxylic acid cycle (TCA). According to our results, we found two important down-regulated genes PDHB and LDHB after *Slc2a6* knockdown. Overall conversion of pyruvate to acetyl CoA and carbon dioxide is catalyzed by PDHB in TCA [27]. LDHB is responsible for the consumption of lactate and regulates the production of pyruvate from catalyzed lactate [28]. Down-regulation of PDHB and LDHB resulted in greater utilization of pyruvate through glycolysis, with increased production and decreased consumption of lactate.

Lactate has been reported to accumulate in the skeletal muscle and blood with the progression of T2DM, which can lead to lactic acidosis [29]. Severe skeletal muscle atrophy was also observed in these patients. Through several investigations have illustrated the underlying mechanism of lactate accumulation inhibiting myogenic differentiation, yet, inconsonant mechanisms have emerged. Lactic acid accumulation significantly inhibited the levels of Akt-mediated myogenic differentiation signaling proteins such as MyoD and myogenin while increasing the levels of AMPK-mediated muscle atrophy signaling pathway [30]. In addition, lactate accumulation induced oxidative stress, enhanced cell-cycle withdrawal, and initiated early myogenic differentiation marker genes Pax7 and Myf5 but delayed late myogenic differentiation stage markers (myogenin and MHC) [31]. Additionally, lactic acid accumulation severely affected p38 MAPK and H3K4me3 signaling in vitro and in vivo, which reducing the expression of Myf5, myogenin, and myosin heavy chain (MHC) and resulted in myogenic differentiation inhibition [32]. Collectively, lactic acid can inhibit myogenic differentiation by changing metabolism-related signaling pathways such as AMPK and AKT through its function as an energy source. Besides that, high-concentration and prolonged lactic acid stimulation caused a metabolic stress and MAPK signaling activating which can inhibit key myogenic gene expression. This study characterizes the negative effect of different concentrations of lactate treatment on myogenic differentiation. However, the mechanism of how lactate interacts with myogenic genes still needs further study.

In this study, knockdown of both *Slc2a6* and LDHB resulted in the accumulation of lactate and markedly decreased the expression of myogenic marker genes, indicating that the *Slc2a6*-LDHB pathway may be an upstream regulator of myogenic differentiation. Therefore, the *Slc2a6*-LDHB pathway can be a key therapeutic target for the management of T2DM-related sarcopenia.

## Conclusions

In summary, we have uncovered a new function of *Slc2a6*, identified LDHB as a *Slc2a6* target gene promoting myogenic differentiation, and demonstrated the inhibitory effect of lactate on myogenesis. Our study broadens the mechanistic understanding of myogenesis and provides potential therapeutic targets for the treatment of T2DM-related sarcopenia.

## Supplementary Information

**Additional file 1: Figure S1**. Characterization of db/db mice and differentiated C2C12 cells. (a) Body weight. (b) Fasting blood glucose level after 12 h of fasting. (c) Representative images of C2C12 myoblasts in the growth medium (GM) and differentiation medium (DM). Scale bar: 100 µm. (d) Western blotting for MyHC, MyoD, and MyoG protein levels after C2C12 myoblast differentiation for 5 d. (e) Heatmap of the twenty most significantly up-regulated genes associated with myogenic differentiation in RNA-seq data. The data are expressed as mean ± SEM, *p < 0.05, **p < 0.01, ***p < 0.001.

**Additional file 2: Figure S2**. Validation of si-*Slc2a6* and si-LDHB function and specificity. (a) The *Slc2a6* mRNA levels in C2C12 cells transfected with si-*Slc2a6*-2 during myoblast differentiation. (b) The mRNA expression of Myf5, MyoD, MyoG, and MyHC in si-NC– or si-*Slc2a6*–2 transfected cells. (c) Western blot analysis for MyHC, MyoD, MyoG, and β-actin. (d). (e) The relative protein levels of MyHC, MyoD and MyoG were normalized by β-actin. *p < 0.05, **p < 0.01, ***p < 0.001 compared with the si-NC group.

**Additional file 3: Figure S3**. (a) Concentration of lactic acid in cell culture medium after *Slc2a6* knockdown. (b) Intracellular pyruvate concentration after LDHB knockdown. *p < 0.05, **p < 0.01, ***p < 0.001 versus si-NC group.

## Data Availability

All supporting data included in the main article and its supplementary files are available from the corresponding author upon request.

## Abbreviations

T2DM: Type 2 diabetes mellitus
GM: Growth medium
DM: Differentiation medium
qPCR: Real-time polymerase chain reaction
Slc2a6: Solute carrier family 2 member 6
MyHC: Myosin heavy chain
MyoD: Myogenic differentiation antigen
MyoG: Myogenin
Myf5: Myogenic factor 5
DEGs: Differentially expressed genes
KEGG: Kyoto encyclopedia of genes and genomes
PDHB: Pyruvate Dehydrogenase E1 Subunit Beta
LDH-A: Lactate dehydrogenase A
LDH-B: Lactate dehydrogenase B
LA: Lactate
