# Supplementary figures and images for: Slc2a6 regulates myoblast differentiation by targeting LDHB
Source: Cell Commun Signal. 2022 Jul 18;20:107. doi: 10.1186/s12964-022-00915-2 (PMC9290262; doi:10.1186/s12964-022-00915-2)

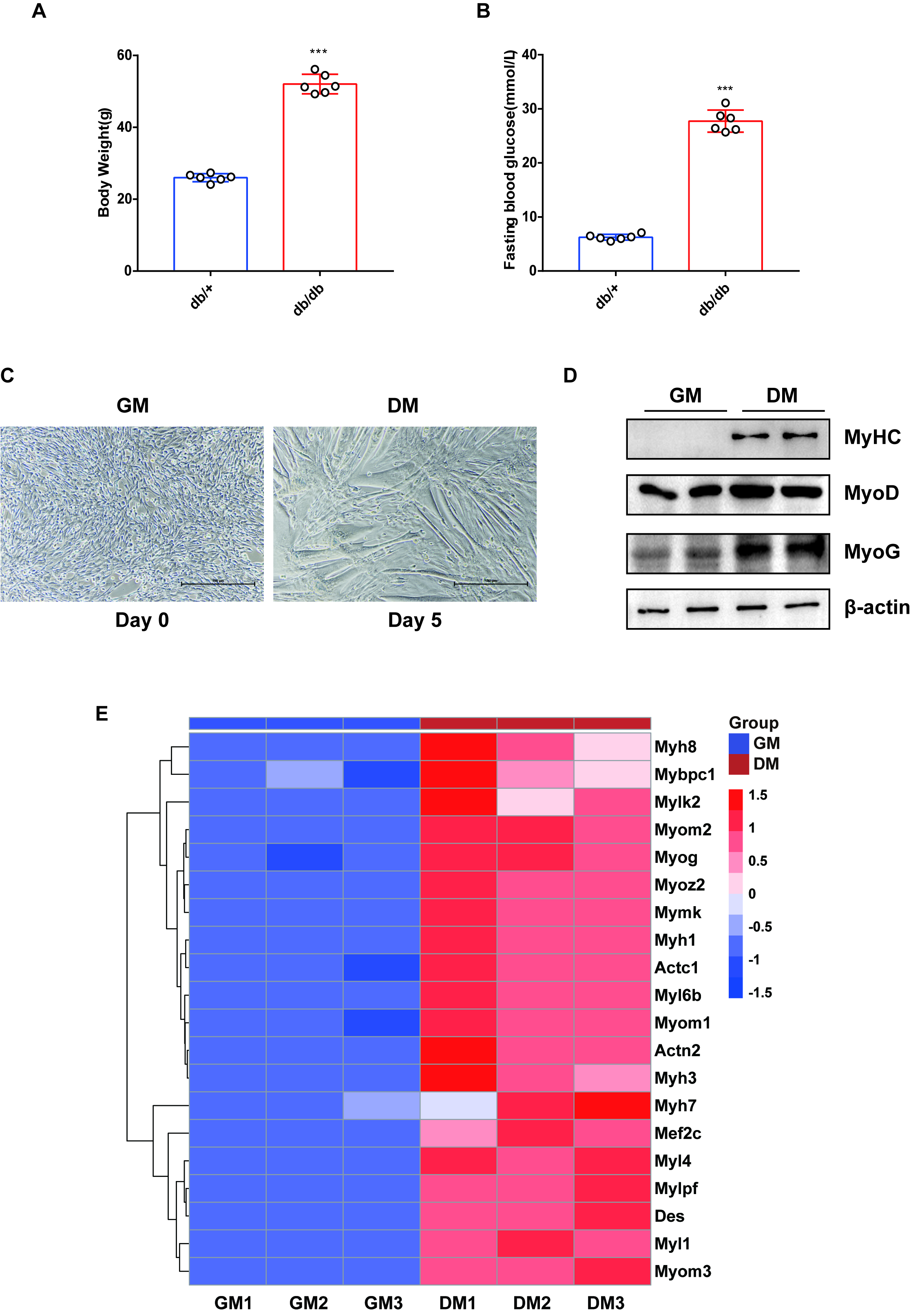

Supplement: Supplementary file 2 — Additional file 1: Figure S1. Characterization of db/db mice and differentiated C2C12 cells. (a) Body weight. (b) Fasting blood glucose level after 12 h of fasting. (c) Representative images of C2C12 myoblasts in the growth medium (GM) and differentiation medium (DM). Scale bar: 100 µm. (d) Western blotting for MyHC, MyoD, and MyoG protein levels after C2C12 myoblast differentiation for 5 d. (e) Heatmap of the twenty most significantly up-regulated genes associated with myogenic differentiation in RNA-seq data. The data are expressed as mean ± SEM, *p < 0.05, **p < 0.01, ***p < 0.001. [file 12964_2022_915_MOESM2_ESM.tif]

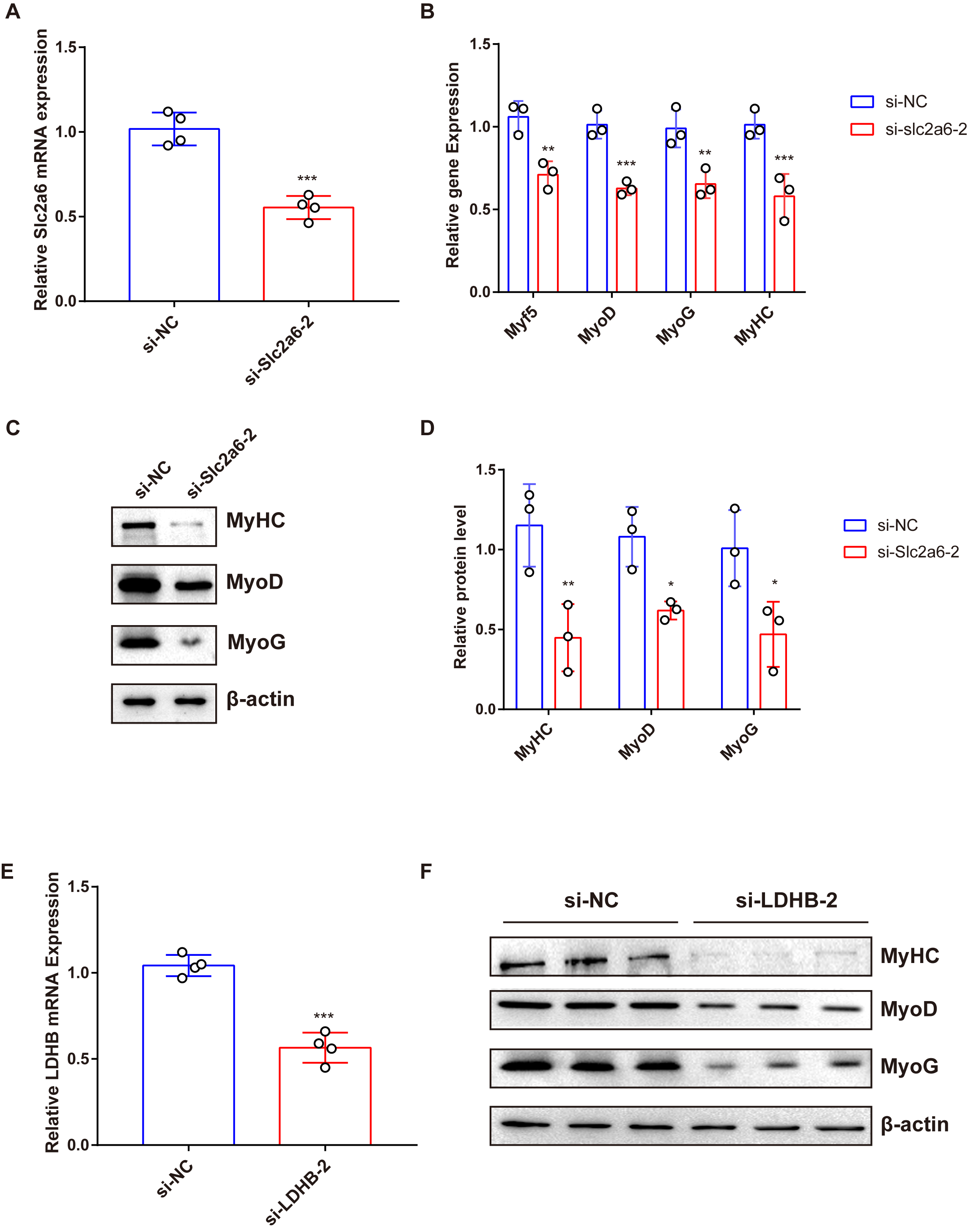

Supplement: Supplementary file 3 — Additional file 2: Figure S2. Validation of si-Slc2a6 and si-LDHB function and specificity. (a) The Slc2a6 mRNA levels in C2C12 cells transfected with si-Slc2a6-2 during myoblast differentiation. (b) The mRNA expression of Myf5, MyoD, MyoG, and MyHC in si-NC– or si-Slc2a6–2 transfected cells. (c) Western blot analysis for MyHC, MyoD, MyoG, and β-actin. (d). (e) The relative protein levels of MyHC, MyoD and MyoG were normalized by β-actin. *p < 0.05, **p < 0.01, ***p < 0.001 compared with the si-NC group. [file 12964_2022_915_MOESM3_ESM.tif]

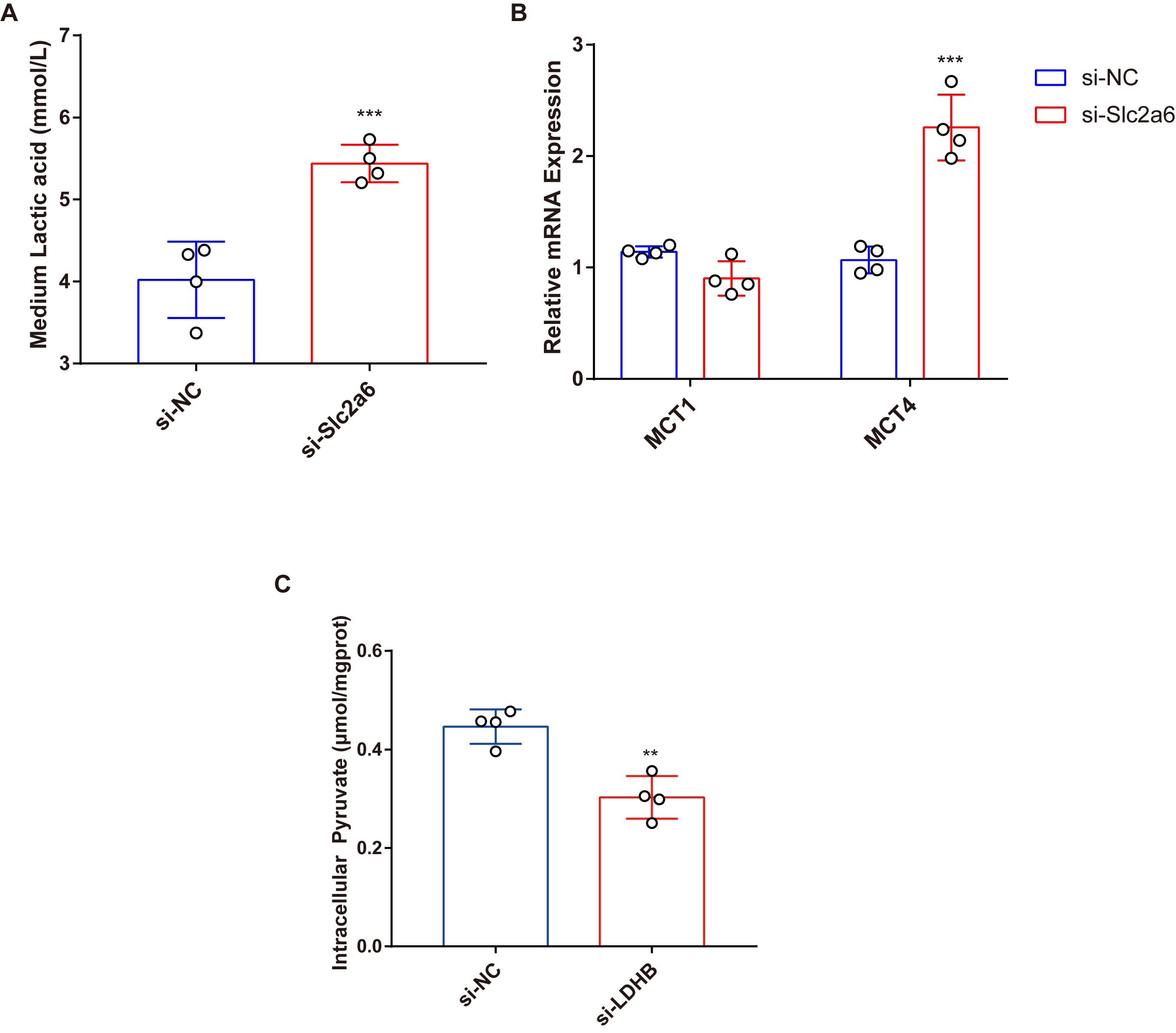

Supplement: Supplementary file 4 — Additional file 3: Figure S3. (a) Concentration of lactic acid in cell culture medium after Slc2a6 knockdown. (b) Intracellular pyruvate concentration after LDHB knockdown. *p < 0.05, **p < 0.01, ***p < 0.001 versus si-NC group. [file 12964_2022_915_MOESM4_ESM.tif]
